# Medial tentorial dural arteriovenous fistula: A rare cause of bithalamic oedema

**DOI:** 10.1016/j.radcr.2022.03.072

**Published:** 2022-04-12

**Authors:** Cyril Dargazanli, Anais Lippi, Nicolas Gaillard

**Affiliations:** 1 Department of Neuroradiology, Montpellier University Hospital Center, Gui de Chauliac Hospital, Montpellier, France; 2 Institut de Génomique Fonctionnelle (IGF), UMR 5203 CNRS – U 1191 INSERM – Univ. Montpellier, Montpellier Cedex 05, France; 3 Department of Neurology, Montpellier University Hospital Center, Gui de Chauliac Hospital, Montpellier, France

**Keywords:** Neuroradiology, Fistula, Dural arteriovenous fistula, Bithalamic oedema, Embolization

## Abstract

A 39-year-old man was admitted after 1 week of headaches and cognitive changes. CT scan showed bithalamic hypodensities, corresponding to bithalamic vasogenic oedema. Punctuate hemorrhage was present, with foci of thalamic enhancement. CT angiography raised the suspicion of arteriovenous shunt. Digital subtraction angiography confirmed a medial falcotentorial dural arteriovenous fistula. Complete embolization was performed using liquid embolic agent. Although tentorial dural fistulas have already been described as a cause of bithalamic oedema and subacute dementia, they are not generally included in pathologies implied in this radiologic pattern.

## Case Presentation

A 39-year-old man without medical history was admitted after 1 week of headaches, cognitive changes with apathy, and loss of language fluence. After initial workup in a psychiatric unit, patient was referred in neurology department. Clinical status quickly worsened with impaired consciousness and akinetic mutism.

**Fig. 1:**
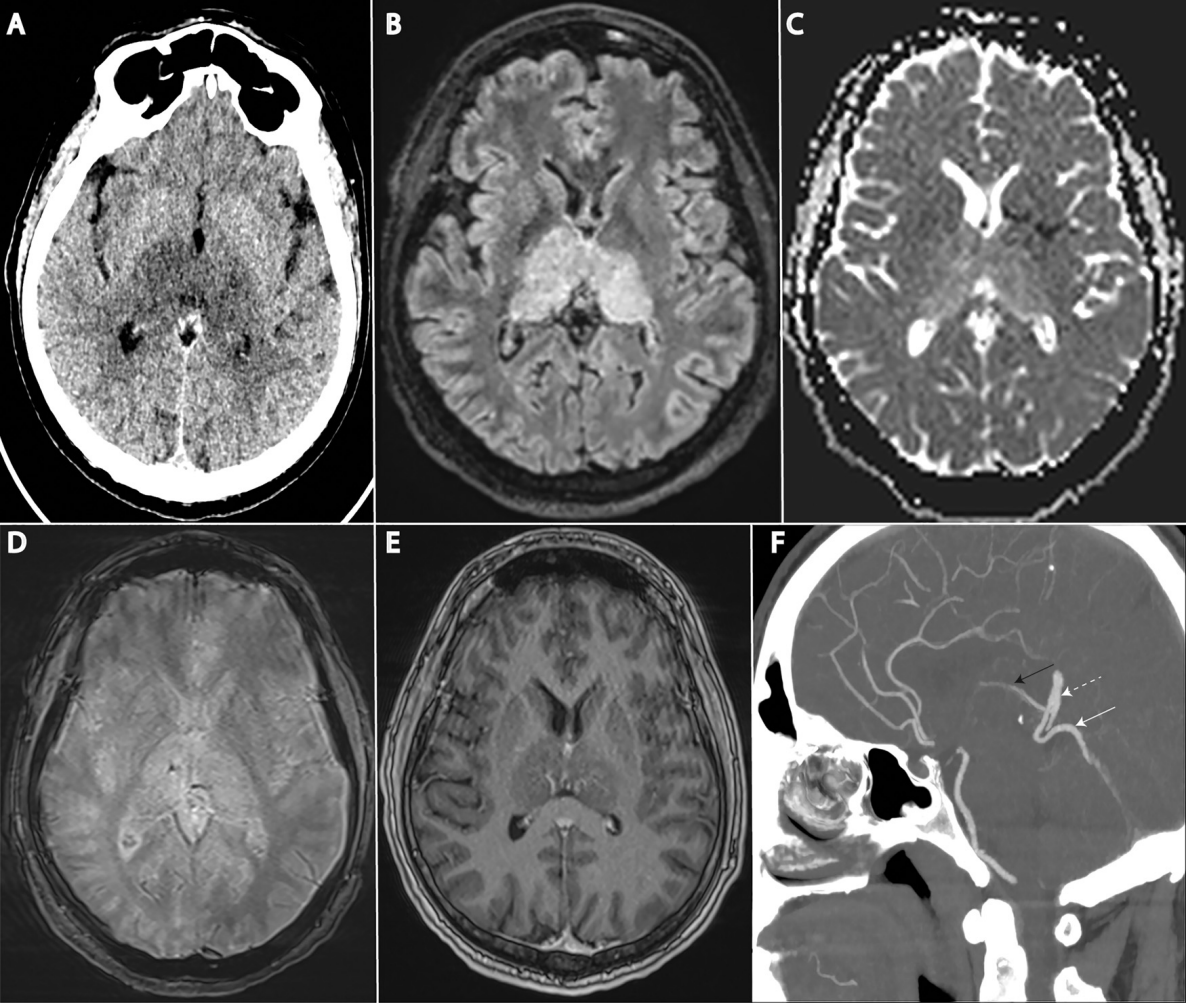
Noninvasive Imaging A: Plain CT scan showing bithalamic symmetrical hypodensities B: Baseline MRI showing bithalamic FLAIR hyperintensities C: Diffusion MRI depicting increased bithalamic apparent diffusion coefficient (ADC) D: Punctuate hemorrhage in right thalamus on T2* E: Gadolinium enhanced sequence showing small foci of bithalamic patchy enhancement F: Arterialized superior vermian vein (white arrow) draining into vein of Galen (dotted white arrow) and subsequently into internal cerebral vein (black arrow).

Admission CT scan showed bithalamic hypodensities (Fig. 1A), corresponding to bithalamic vasogenic oedema on MRI (Fig. 1B,C). Subtle punctuate petechial hemorrhage was present (Fig. 1D), and enhanced MRI showed small foci of thalamic patchy enhancement (Fig. 1E), without obvious deep venous thrombosis. Lumbar puncture showed slight hyperproteinorachia (0.69 g/L), without increased cellularity or abnormal cells.

CT angiography raised the suspicion of arteriovenous shunt given the opacification of superior vermian vein at the arterial phase (Fig. 1F).

**Fig. 2:**
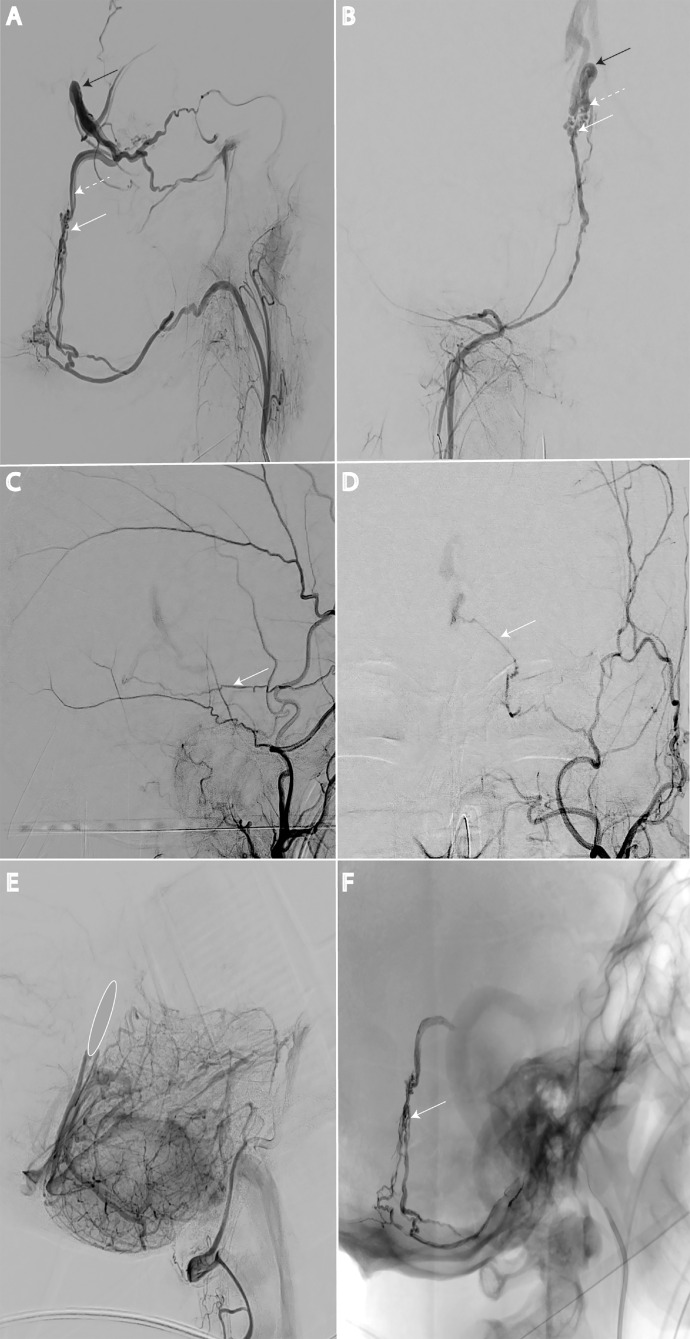
Digital Subtraction Angiography (DSA) A and B: Lateral (A) and anteroposterior (B) arterial phase showing the fistulous plaque (white arrow), the arterialized superior vermian vein (dotted white arrow) and vein of Galen (black arrow) C and D: Small contribution of the medial tentorial branch from the left middle meningeal artery (white arrow) E: Lateral venous phase of left vertebral artery injection showing filling defect of the anterior part of straight sinus, related to deep venous thrombosis F: Post embolization non-substracted image showing liquid-embolic cast in the arterial feeders, in the fistulous plaque, but away from the vein of Galen.

Digital subtraction angiography (DSA) was decided at this time, confirming a medial falcotentorial dural arteriovenous fistula (dAVF). Arterial supply was mainly from both falx cerebelli branches of the posterior meningeal arteries coming from the neuromeningeal trunks of the anterior pharyngeal arteries (APA, Fig. 2A, B), with a faint accessory supply from the medial tentorial branch of the left middle meningeal artery (Fig. 2C, D).

Anterior segment of the straight sinus was thrombosed (Fig. 2E), explaining the venous drainage into the superior vermian vein and the vein of Galen.

Complete embolization was performed using liquid embolic agent (ethylene-vinyl copolymer suspended in dimethyl sulfoxide and opacified with tantalum [Onyx, eV3]) through the left APA route (Fig. 2F).

Patient's cognition improved over the next month, with spontaneous mobilization 8 days after the embolization and complete normalization of vigilance.

However, patient was discharged in a rehabilitation center regarding persistence of severe comprehension disorders and aphasia.

MRI performed at 3 months showed complete resolution of thalamic anomalies.

## Discussion

A broad list of conditions (vascular, metabolic, infectious or neoplastic) is associated with bithalamic oedema. Although tentorial dural fistulas have already been described as a cause of bithalamic oedema and subacute dementia [1,2], they are not generally included in pathologies implied in this classical radiologic pattern [3]. Diagnostic may be difficult on noninvasive imaging, and these rare dAVF are potentially overlooked. Moreover, non-hemorrhagic presentation (thalamic dementia) may delay angiographic diagnosis, with a mean duration of symptoms at the time of diagnosis around 3 months [1]. Right diagnostic is of paramount importance, and may avoid inadequate brain biopsy.

Tentorial dural arteriovenous fistulas may be classified into 6 types on the basis of shunt location, associated sinus and direction of venous outflow [4]. Arterial feeders for the straight sinus/medial falcotentorial subtype are mainly the posterior meningeal artery, occipital artery or tentorial artery. Both endovascular and surgical procedures may be efficiently performed to cure these aDVF.

In conclusion, medial tentorial dAVF should be evocated in patients with unexplained rapidly progressive cognitive impairment and bithalamic oedema, and complete DSA should be performed in these situations.

## Patient consent

Informed consent for publication of this case was obtained from the patient.
